# Variations in Energy Metabolism Precede Alterations in Cardiac Structure and Function in Hypertrophic Preconditioning

**DOI:** 10.3389/fcvm.2020.602100

**Published:** 2020-12-11

**Authors:** Jian Wu, Jing Lu, Jiayuan Huang, Jieyun You, Zhiwen Ding, Leilei Ma, Fangjie Dai, Ran Xu, Xuan Li, Peipei Yin, Gang Zhao, Shijun Wang, Jie Yuan, Xiangdong Yang, Junbo Ge, Yunzeng Zou

**Affiliations:** ^1^Shanghai Institute of Cardiovascular Diseases, Zhongshan Hospital and Institutes of Biomedical Sciences, Fudan University, Shanghai, China; ^2^Key Laboratory of Guangdong Laboratory Animals, Guangdong Laboratory Animals Monitoring Institute, Guangzhou, China; ^3^Department of Cardiovascular Medicine, Shanghai East Hospital, Tongji University School of Medicine, Shanghai, China

**Keywords:** cardiac function, cardiac hypertrophy, hypertrophic preconditioning, metabolism, pressure overload

## Abstract

Recent studies have unveiled that myocardial hypertrophic preconditioning (HP), which is produced by de-banding (De-TAC) of short-term transverse aortic constriction (TAC), protects the heart against hypertrophic responses caused by subsequent re-constriction (Re-TAC) in mice. Although cardiac substrate metabolism is impaired in heart failure, it remains unclear about the role of HP-driven energetics in the development of cardiac hypertrophy. Here, we investigated energy metabolism, cardiac hypertrophy, and function following variational loading conditions, as well as their relationships in HP. Male C57BL/6J mice (10–12 weeks old) were randomly subjected to Sham, HP [TAC for 3days (TAC 3d), de-banding the aorta for 4 days (De-TAC 4d), and then re-banding the aorta for 4 weeks (Re-TAC 4W)], and TAC (TAC for 4 weeks without de-banding). Cardiac echocardiography, hemodynamics, and histology were utilized to evaluate cardiac remodeling and function. The mRNA expression levels of fetal genes (*ANP* and *BNP*), glucose metabolism-related genes (*glut4, pdk4*), and fatty acid oxidation-related genes (*mcad, pgc1*α, *mcd, ppar*α) were quantitated by real-time quantitative PCR. Activation of hypertrophy regulators ERK1/2, a metabolic stress kinase AMP-activated protein kinase (AMPK), and its downstream target acetyl-coA carboxylase (ACC) were explored by western blot. Compared with TAC 4W mice, Re-TAC 4W mice showed less impairment in glucose and fatty acid metabolism, as well as less cardiac hypertrophy and dysfunction. Moreover, no significant difference was found in myocardial hypertrophy, fibrosis, and cardiac function in TAC 3d and De-TAC 4d groups compared with Sham group. However, *glut4, pdk4, mcad, pgc1*α, *mcd*, and *ppar*α were all decreased, while AMPK and ACC were activated in TAC 3d and returned to Sham level in De-TAC 4d, suggesting that the change in myocardial energy metabolism in HP mice was earlier than that in cardiac structure and function. Collectively, HP improves energy metabolism and delays cardiac remodeling, highlighting that early metabolic improvements drive a potential beneficial effect on structural and functional restoration in cardiac hypertrophy.

## Introduction

Heart failure (HF) is a leading cause of death, threatening human health worldwide (1). Left ventricular (LV) hypertrophy is an adaptive alteration of the heart in response to increased mechanical overload, *per se* serving as an independent risk factor of major cardiac events (2, 3). Clinical studies on hypertensive patients and basic studies using animals with transverse aortic constriction (TAC) indicate that sustained pressure overload causes the transition from compensated to decompensated cardiac hypertrophy with the eventual progression to HF (4). If pressure overload is withdrawn timely, regression of cardiac remodeling is accompanied with functional improvement (5). Intriguingly, similar to ischemic preconditioning whereby a brief episode of ischemia increases cardiac resistance to subsequent prolonged ischemia, hypertrophic preconditioning (HP) also exists in the heart (6, 7). Using a unique *in vivo* model of HP, we and others sequentially imposed, removed, and re-imposed pressure overload on the mouse heart through TAC, de-banding the aorta (De-TAC), and then re-banding the aorta (Re-TAC). It is found that the heart was protected from the subsequent pressure overload induced by Re-TAC (6, 7). Although the discovery of HP would convincingly explain diverse extent of cardiac hypertrophy shown in hypertensive patients and enlighten us with new strategy to blunt the initiation of cardiac hypertrophy, the underlying mechanism remains largely unknown.

It is well-established that LV hypertrophy and HF are associated with metabolic abnormalities (8). Pressure overload forces a shift of reliance on glucose instead of fatty acids to provide energy for the heart (9, 10). The prominent metabolic changes result in impaired cardiac energetics, which precedes manifestation of cardiac dysfunction and LV hypertrophy (8, 11). Although many of these metabolic changes are due to an aberration of transcriptional expression level of metabolic enzymes, it is only recently found that this abnormality of metabolic flexibility is reversible, for normalization of cardiac energy metabolism and LV hypertrophy precedes recovery of LV function, in HF mice with De-TAC and in HF patients with left ventricular assist device (LVAD)-induced mechanical unloading (1, 5). Moreover, considering HP renders the heart resistant to hypertrophy, it would have clinical implications for early detection and prevention of LV hypertrophy and HF, through examination of correlation between metabolic changes, LV function, and hypertrophy. Therefore, we in this study used a mouse model of HP to determine the temporal and causal relationships of these changes.

## Materials and Methods

### Experimental Animals

Male C57BL/6J mice (12–13 weeks old, purchased from the Department of Laboratory Animal Sciences, Fudan University, Shanghai, China) were enrolled in this study. The animals were housed under controlled conditions (24 ± 2°C, 12-h dark/12-h light cycle, *ad libitum* access to normal chow and water). All animal experiments conformed to the Guide for the Care and Use of Laboratory Animals (No. 85-23, revised 1996; National Institutes of Health, Bethesda, MD, USA). The study protocol was approved by the Animal Care and Use Committee of Zhongshan Hospital, Fudan University.

The mice were randomly assigned to Sham (*n* = 10), TAC (*n* = 15), HP (*n* = 40) groups. For HP mice, serial assessments described below were performed at Baseline (1 day before operation), 3 days after TAC (TAC 3d), 4 days after aortic de-banding (De-TAC 4d), and 4 weeks after re-banding (Re-TAC 4W) (Supplementary Figure 1). The similar thoracotomies were also performed in Sham and TAC mice to minimize the possible confounding effects of repeated surgical injuries. At each time point, at least three mice were randomly selected for histology, quantitative real-time PCR, and Western blot analyses.

### TAC, De-TAC, and Re-TAC

TAC surgery was performed as we described previously (7, 12). In brief, mice were anesthetized by 2% isoflurane; the aortic arch was isolated and secured against a 27-gauge needle with a 6-0 silk ligature, which was promptly removed to form an aortic constriction. Meloxicam (0.13 mg each) was administered for analgesia. Sham mice underwent similar operation without tying the ligature around the aorta.

Three days after TAC, a second thoracotomy was given through the original incision site to de-band the aorta (De-TAC). An additional 4 days later, mice were subjected to a third thoracotomy to re-band the aorta (Re-TAC).

### Echocardiography

Cardiac structure and function under 1.5% isoflurane (7, 13) were evaluated by a Vevo2100 high-frequency ultrasound system with a 30-MHz transducer (VisualSonics, Toronto, ON, Canada). The echocardiographic assessment had good intra- and inter-observer agreement as reported in our previous studies (4, 7, 14, 15). Peak flow velocity at the aortic banding site (PVb, for prediction of pressure gradients, see Supplementary Figure 2), left ventricle posterior wall thickness in diastole (LVPWTd) and systole (LVPWTs), left ventricular end-diastolic (LVEDD) and end-systolic (LVESD) dimensions, and left ventricular ejection fraction (LVEF) and fractional shortening (LVFS) were acquired.

### Invasive Hemodynamics

After echocardiography, LV hemodynamics were evaluated as we described previously (4, 7). Briefly, under 2% isoflurane, a micromanometer (Millar 1.4F, SPR 835; Millar Instruments, Houston, TX) was inserted into the right common carotid artery and then advanced into the LV for measurement of end-systolic and end-diastolic pressure (LVESP and LVEDP), maximal contraction, and relaxation velocity (Max dp/dt and Min dp/dt).

### Histology

Mice were euthanized after hemodynamic measurements. Hearts were excised, rinsed in saline, and heart weight-to-body weight ratios are compared (HW/BW). The LV tissue was separated into several parts longitudinally and stored at −80°C for further analysis after removing the right ventricular free wall. The hearts were stained with hematoxylin and eosin (H&E) for assessment of cardiomyocyte size or with Masson trichrome for cardiac fibrosis. Digital photographs were taken using an image analysis system (KF-TB-400, Konfoong biotech international, Ningbo, China).

### Quantitative Real-Time PCR

LV tissue samples were isolated and homogenized in TRIzol Reagent (ThermoFisher Scientific, Carlsbad, CA, USA). The RNA concentration was assessed by spectrophotometry (NanoDrop, ThermoFisher Scientific, Carlsbad, CA, USA). Equivalent amounts (2 μg) of purified RNA were used as a template to synthesize cDNA by the Hifair III cDNA Synthesis SuperMix (Yeasen, Shanghai, China). Quantitative real-time (qRT)-PCR was performed using QuantStudio 6 Flex Real-Time PCR System with ChamQ Universal SYBR qPCR Master Mix (Vazyme biotech, Nanjing, China). Relative mRNA expression levels of target genes were normalized to the amount of the constitutively expressed housekeeping gene β*-actin* (*Actb*). Based on previous studies (4, 5, 7, 8, 16), relative gene expression levels of hypertrophy markers such as atrial natriuretic peptide and brain natriuretic peptide (*anp* and *bnp*), fibrosis such as collagen type I alpha 1 (*col1*α*1*) and collagen type 3 alpha 1 (*col3*α*1*), glucose metabolism such as pyruvate dehydrogenase kinase 4 and glucose transporter type 4 (*glut4* and *pdk4*), and fatty acid metabolism such as medium-chain acyl-CoA dehydrogenase, malonyl-CoA decarboxylase, peroxisome proliferator-activated receptor α, and peroxisome proliferator-activated receptor gamma coactivator 1α (*mcad, mcd, ppar*α, and *pgc1*α) were analyzed using the 2^−ΔΔCt^ method. The nucleotide sequences of primers used are shown in Supplementary Table 1.

### Western Blot Analysis

LV tissue was homogenized in RIPA lysis buffer (Beyotime Biotechnology, Shanghai, China), electrophoresed in 8% or 10% polyacrylamide gels, and transferred to a PVDF membrane (Millipore, Billerica, MA, USA). Membranes were blocked with 5% bovine serum albumin, followed by incubation with appropriate primary antibodies: anti-extracellular signal-regulated kinase (ERK1/2, #9102), anti-phosphorylated (p-)ERK1/2 (p-ERK1/2, #9101A), anti-p-AMP-activated protein kinase α (p-AMPK, #2535), and anti-p-acetyl-coA carboxylase (p-ACC, #3661). Those primary antibodies were purchased from Cell Signaling Technology (Danvers, MA, USA). The blotted membranes were incubated with horseradish peroxidase-conjugated rabbit secondary antibody (Kang-Chen Biotechnology, Shanghai, China), detected with chemiluminescence reagents (catalog RPN2106, GE Healthcare, Piscataway, NJ, USA), visualized using a LAS-3000 chemiluminescence detection system (FUJIFILM, Kanagawa, Japan), and analyzed with the ImageJ software (National Institutes of Health, Bethesda, MD, USA).

### Statistics

Data are expressed as the means ± SEM. Statistical evaluations were performed using the software SPSS 15.0 (SPSS Inc., Chicago, IL, USA). Multiple comparisons were conducted by one-way ANOVA with the Student-Newman-Keuls (S-N-K) test. *P* < 0.05 was considered statistically significant.

## Results

### HP Attenuates LV Hypertrophy in Pressure Overload Hearts

**Figure 1:**
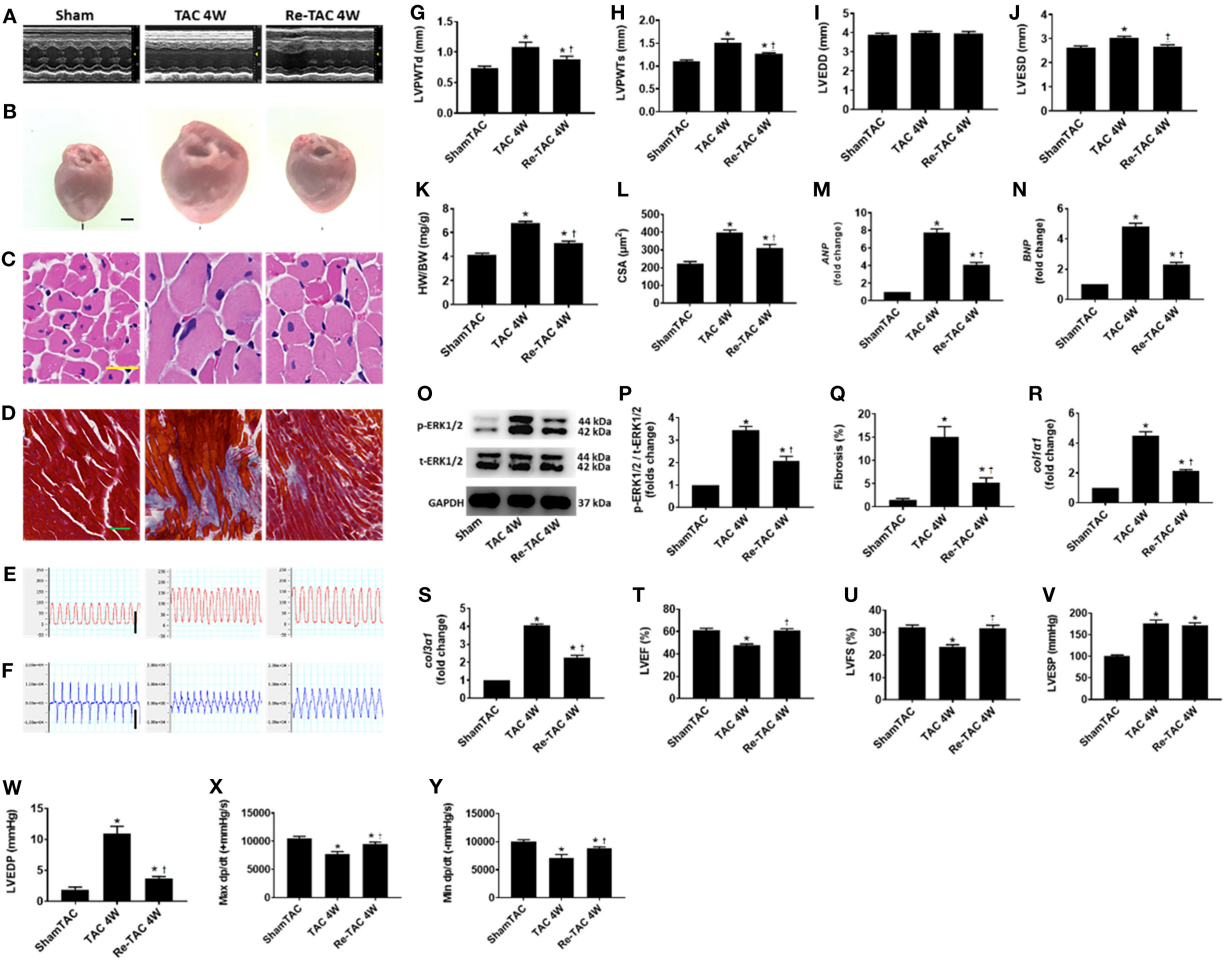
Hypertrophic preconditioning improves cardiac morphological changes and function in pressure overload hearts. **(A)** M-mode echocardiography from Sham, TAC 4W, and Re-TAC 4W mice. **(B)** Gross appearance of hearts. Scale bar = 1 mm. **(C)** Cardiomyocytes stained by H&E. Scale bar = 20 μm. **(D)** Cardiac fibrosis stained by Masson's trichrome. Scale bar = 50 μm. Representative images of **(E)** LV pressure and **(F)** contraction and relaxation velocity (dp/dt). Scale bar = 100 mmHg for pressure, scale bar = 10,000 mmHg/s for dp/dt. **(G,H)** LV end-diastolic (LVPWTd) and end-systolic (LVPWTs) posterior wall thickness, respectively. **(I,J)** LV end-diastolic (LVEDD) and end-systolic (LVESD) dimension, respectively. **(K)** Heart weight-to-body weight ratio (HW/BW). **(L)** Cross-sectional area (CSA) of cardiomyocytes. **(M,N)** Real-time quantitative PCR analyses for the gene expression of atrial natriuretic peptide (*ANP*) and brain natriuretic peptide (*BNP*), respectively. Representative images **(O)** and densitometric assessment **(P)** of Western blot for ERK1/2 activation. **(Q)** Quantification of collagen staining expressed as % area. Gene expression of makers of cardiac fibrosis **(R)** collagen type I alpha 1 (*col1*α*1*) and **(S)** collagen type 3 alpha 1 (*col3*α*1*). Echocardiographic measurements of **(T)** LV ejection fraction (LVEF) and **(U)** LV fractional shortening (LVFS). Invasive hemodynamic assessment of **(V,W)** LV end-systolic (LVESP) and end-diastolic (LVEDP) pressure, respectively. **(X,Y)** Maximum (Max dp/dt) and minimum (Min dp/dt) value of LV dp/dt, respectively. *P < 0.05 vs. Sham TAC; ^†^*P* < 0.05 vs. TAC 4W.

Decompensated LV hypertrophy in response to chronic pressure overload is characterized by thickening of the heart muscle and dilation of LV chamber (7, 17). Compared with Sham mice, TAC 4W mice showed decompensated cardiac hypertrophy evidenced by a striking increase of HW/BW, LV wall thickness, inner dimension, fibrosis, and cardiomyocyte size (Figure 1). Re-TAC 4W mice showed that HP treatment significantly mitigated the morphological changes compared with TAC 4W mice, although still showed moderate cardiac hypertrophy compared with Sham mice (Figure 1).

The hypertrophic response to pressure overload are underlined with reprogramming of fetal genes and activation of hypertrophic signaling effectors (12, 17). We therefore measured cardiac mRNA and protein levels of markers of LV hypertrophy and failure. Compared with Sham mice, *ANP* and *BNP* in TAC 4W mice are overtly up-regulated, as well as p-ERK1/2 activation (Figure 1). HP in Re-TAC 4W mice notably compromised the hypertrophic response at a molecular level (Figure 1).

### HP Improves LV Function in Pressure Overload Hearts

As we and others previously reported, 4 weeks of pressure overload results in cardiac dysfunction (4, 12, 18). To determine whether HP restored cardiac function, mice in this study were subjected to echocardiography and invasive hemodynamic analysis. TAC 4W mice showed a prominent reduction in LVEF, LVFS, Max dp/dt, and Min dp/dt and an increase in LVEDP, compared with Sham mice. Intriguingly, Re-TAC 4W mice presented an improvement in the aforementioned indexes (Figure 1).

### HP Improves Substrate Utilization and Alleviates Metabolic Changes in Pressure Overload Hearts

**Figure 2:**
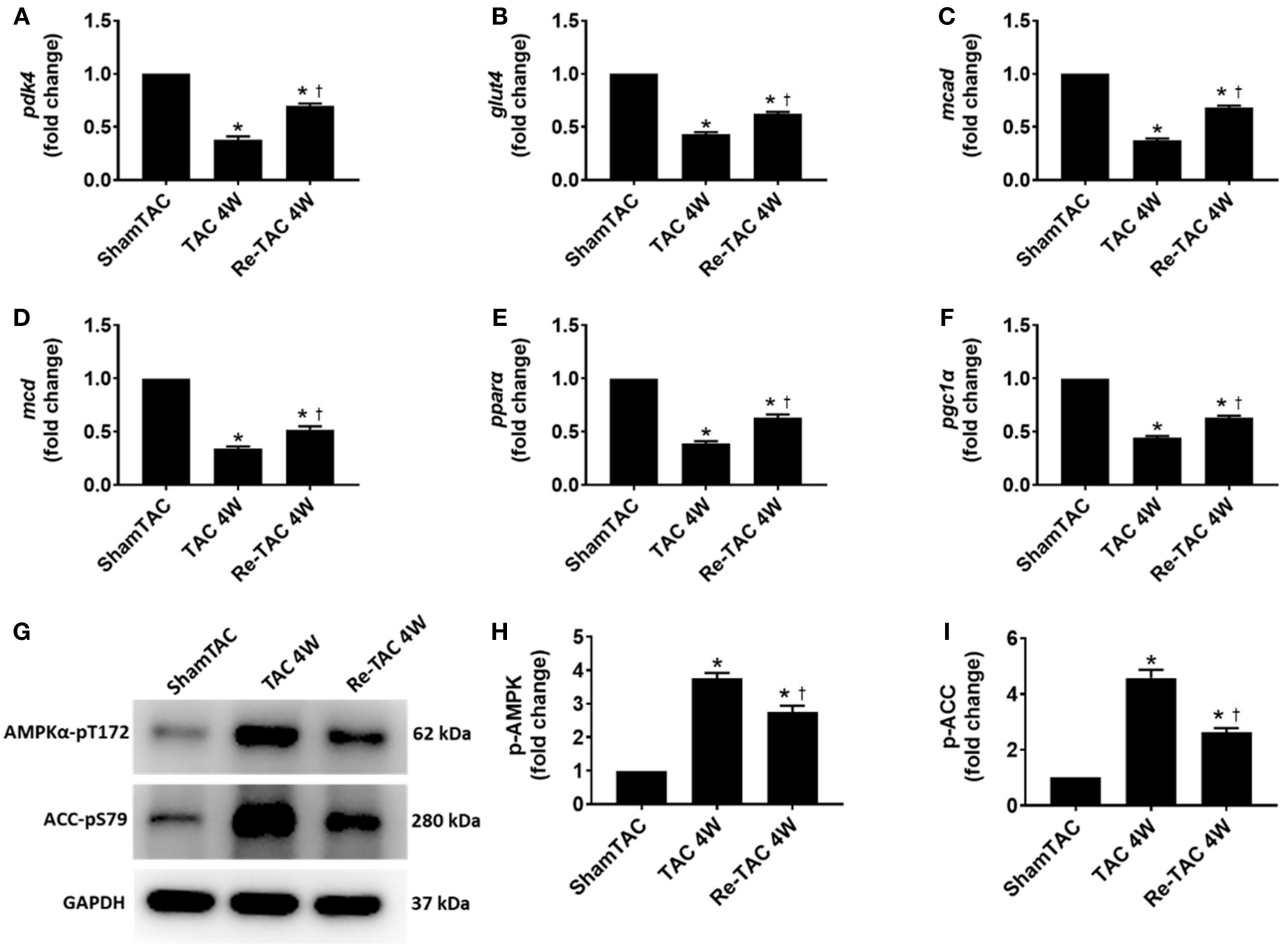
Hypertrophic preconditioning improves substrate utilization in pressure overload hearts. Real-time quantitative PCR analyses for the gene expression of **(A)** pyruvate dehydrogenase kinase 4 (*pdk4*), **(B)** glucose transporter type 4 (*glut4*), **(C)** medium-chain acyl-CoA dehydrogenase (mcad), **(D)** malonyl-CoA decarboxylase (*mcd*), **(E)** peroxisome proliferator-activated receptor α (*ppar*α), and **(F)** peroxisome proliferator-activated receptor gamma coactivator 1α (*pgc1*α). Representative images **(G)** and densitometric assessment **(H,I)** of cardiac phosphorylated AMP-activated protein kinase (p-AMPK) and phosphorylated acetyl-CoA carboxylase (p-ACC) as acquired by Western blot analysis. *P < 0.05 vs. Sham TAC; ^†^*P* < 0.05 vs. TAC 4W.

Sustained pressure overload is associated with alterations at the transcriptional level of metabolic enzymes that is detrimental to cardiac energetics (5). To interrogate whether these changes are permanent, the transcript levels of typical genes associated with metabolism of glucose (*glut4* and *pdk4*) and fatty acid (*mcad, mcd, pgc1*α, and *ppar*α) were measured. They were considerably reduced in TAC 4W hearts but were mostly recovered to the sham-operated level in Re-TAC 4W hearts (Figure 2).

To underpin the correlation between mRNA and protein expression levels, we investigated the activation of AMPK, a key metabolic stress kinase, at a post-translational level (19). We found that AMPK was robustly activated in TAC 4W mice, which was recapitulated by its downstream target ACC (Figure 2), whereas Re-TAC 4W mice showed less activation in AMPK and ACC.

### Serial Assessment in HP-Treated Mice Reveals Delayed Changes in LV Morphology

**Figure 3:**
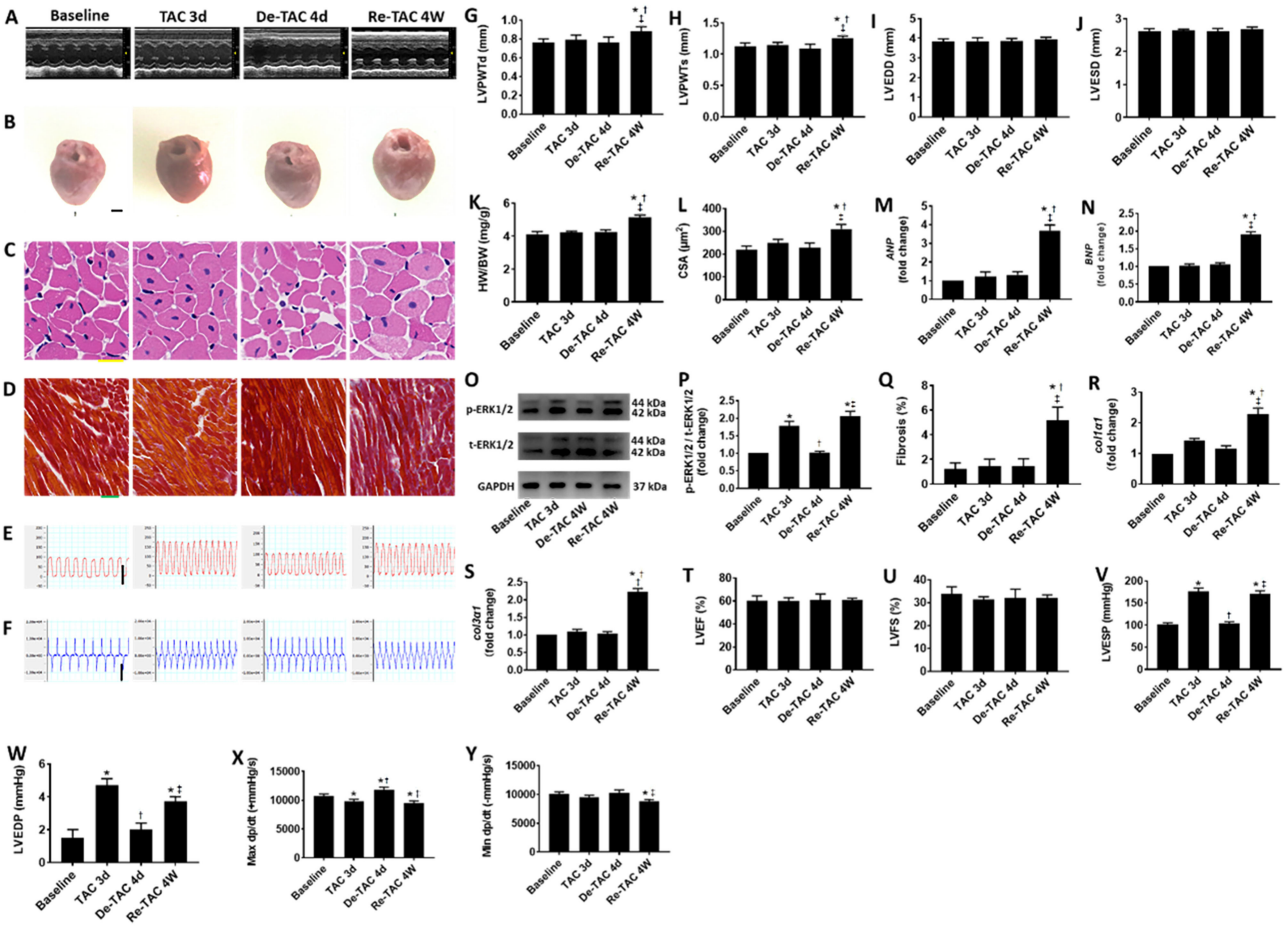
Serial assessment of cardiac morphological and functional changes in HP murine hearts. **(A)** M-mode echocardiography of LVs at baseline, 3 days after TAC (TAC 3d), 4 days after de-banding (De-TAC 4d), and 4 weeks after re-banding (Re-TAC 4W). **(B)** Gross appearance of hearts. Scale bar = 1 mm. **(C)** Cardiomyocytes stained by H&E. Scale bar = 20 μm. **(D)** Cardiac fibrosis stained by Masson's trichrome. Scale bar = 50 μm. Representative images of **(E)** LV pressure and **(F)** dp/dt. Scale bar = 100 mmHg for pressure, scale bar = 10,000 mmHg/s for dp/dt. **(G)** LVPWTd. **(H)** LVPWTs. **(I)** LVEDD. **(J)** LVESD. **(K)** HW/BW. **(L)** CSA of cardiomyocytes. **(M,N)** Real-time quantitative PCR analyses for the gene expression of *ANP* and *BNP*, respectively. Representative images **(O)** and densitometric assessment **(P)** of Western blot for ERK1/2 activation. **(Q)** Quantification of collagen staining expressed as % area. Gene expression of makers of cardiac fibrosis **(R)** *col1*α*1* and **(S)** *col3*α*1*. Echocardiographic measurements of **(T)** LVEF and **(U)** LVFS. Invasive hemodynamic assessment of **(V)** LVESP, **(W)** LVEDP, **(X)** Max dp/dt, **(Y)** Min dp/dt. *P < 0.05 vs. Baseline; ^†^*P* < 0.05 vs. TAC 3d; ^‡^*P* < 0.05 vs. De-TAC 4d.

To investigate the process of LV hypertrophy in the murine HP model, serial evaluations were performed in Baseline, TAC 3d, De-TAC 4d, and Re-TAC 4W mice. We found that HW/BW, LV wall thickness, inner dimension, fibrosis, and cardiomyocyte size were comparable in Baseline mice, TAC 3d mice, and De-TAC 4d mice. Only Re-TAC 4W mice showed signs of moderate cardiac hypertrophy compared with the above groups (Figure 3).

At mRNA and protein levels, Baseline mice and De-TAC 4d mice demonstrated similar scenarios without hypertrophic characteristics. Intriguingly, TAC 3d mice displayed increased ERK1/2 phosphorylation, although *ANP* and *BNP* showed an increasing trend without statistical significance (Figure 3). Re-TAC 4W mice showed a moderate increase of *ANP* and *BNP* expression and p-ERK1/2 activation.

### Serial Assessment in HP-Treated Mice Reveals Delayed Changes in Cardiac Function

To explore LV function closely in the murine HP model, serial echocardiography and invasive hemodynamic analyses were performed in Baseline, TAC 3d, De-TAC 4d, and Re-TAC 4W mice. LVEF and LVFS were not significantly different for mice at all time points (Figure 3), whereas invasive hemodynamics showed a transient reduction in cardiac function in TAC 3d hearts especially manifested in LVEDP and Max dp/dt, following a recovery in De-TAC 4d hearts. Cardiac function reflected by LVEDP, Max dp/dt, and Min dp/dt was moderately impaired after Re-TAC 4W (Figure 3). Taken together, in most instances, these data demonstrated that HP delayed the onset of functional decompensation in pressure overload hearts.

### Serial Assessment in HP-Treated Mice Reveals Early Changes in Cardiac Energy Metabolism

**Figure 4:**
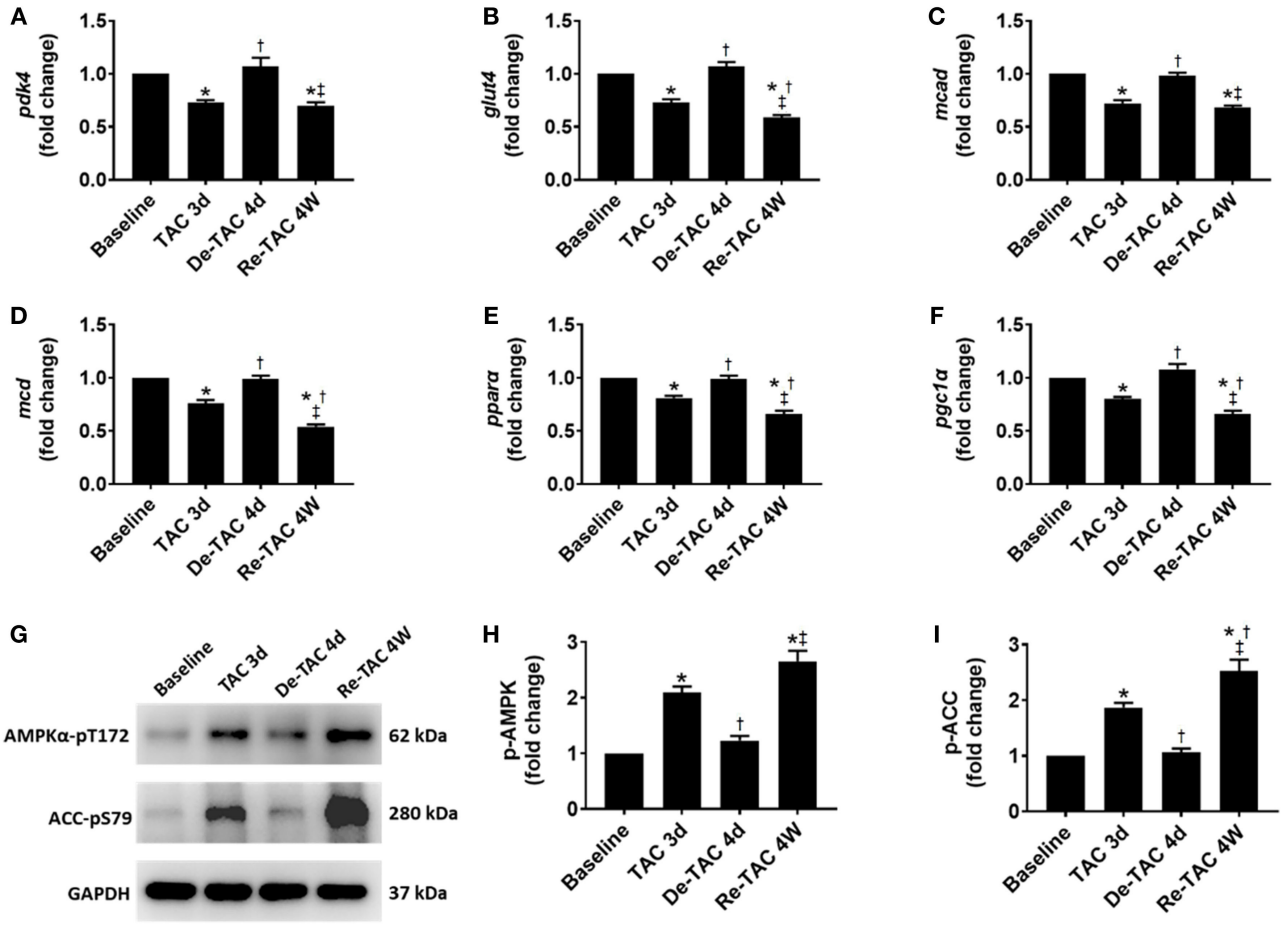
Serial assessment of substrate utilization in HP murine hearts. Real-time quantitative PCR analyses for the gene expression of **(A)** *pdk4*, **(B)** *glut4*, **(C)** mcad, **(D)** *mcd*, **(E)** *ppar*α, and **(F)** *pgc1*α. Representative images **(G)** and densitometric assessment **(H,I)** of cardiac p-AMPK and p-ACC as acquired by Western blot analysis. *P < 0.05 vs. Baseline; ^†^*P* < 0.05 vs. TAC 3d; ^‡^*P* < 0.05 vs. De-TAC 4d.

All genes involved in the metabolism of glucose (*glut4* and *pdk4*) and fatty acid (*mcad, mcd, pgc1*α, and *ppar*α) were downregulated in TAC 3d hearts when compared to Baseline mice. The gene expression was elevated to Baseline levels in De-TAC 4d hearts, then was suppressed again in Re-TAC 4W hearts, although *pdk4* and *mcad* were downregulated to a lesser extent compared with other metabolism markers (Figure 4).

Consistent with our gene expression data, at a post-translational level, AMPK and ACC were considerably activated in TAC 3d mice, recessed to Baseline level, and fueled again in Re-TAC 4W mice (Figure 4), suggesting improved cardiac energetic status precedes morphological and functional recovery in pressure overload hearts.

## Discussion

This study, to the best of our knowledge, for the first time examined the temporal and causal relationships among cardiac morphological, functional, and metabolic changes in a murine model of HP. We observed HP-improved cardiac energetics, morphology, and function effectively in the pressure overload heart. We also identified that short-term TAC treatment induced swift changes in levels of metabolites associated with energy provision and sensing, which varied in accordance with loading conditions. The metabolic changes occurred at early stages before the development of cardiac hypertrophy and dysfunction, which may serve as a sensitive indicator and predisposing factor for cardiac hypertrophy.

LV hypertrophy and HF are tightly associated with metabolic abnormalities (8). Hypertrophied hearts switch from mainly using fatty acids to preferential utilization of glucose for energy production (9). The switch compromises the capacity of cardiac mitochondrial ATP production, contributing to exacerbation of LV hypertrophy and dysfunction (5, 11). On the other hand, preservation of fatty acid oxidation prevents the shift of substrate preference, ameliorates cardiomyocyte hypertrophy, and maintains cardiac function (9). It is thus proposed that maladaptive metabolic remodeling precedes, triggers, and preserves detrimental contractile dysfunction in heart failure progression (20). Nevertheless, only recently, by using pressure overload models and hypertrophy regression models, as well as in patients with LVAD-induced mechanical unloading, it has been evident that metabolic deficit is an early event that contributes to the onset of LV hypertrophy and progression of HF, rather than a consequence of HF (1, 5, 11). In the present study, we with a HP *in vivo* model further demonstrated that the attenuation of LV hypertrophy and regression of function is associated with early improvements in cardiac metabolism, which corroborates the concept that early interventions in metabolic reprogramming are critical for subsequent morphological and functional recovery.

AMPK, a serine-threonine kinase, coordinates anabolic and catabolic processes as a metabolic stress sensor and thus endogenously reflects cardiac myocardial energetic status (19, 21). AMPK and its downstream target ACC is activated in TAC hearts (22) but normalized in De-TAC hearts (5). We further showed that the activation of AMPK and ACC was attenuated by HP and varied in concert with changes in loading conditions of the heart. Intriguingly, a recent study using 5-aminoimidazole-4-carboxamide ribonucleotide (AICAR) to activate AMPK transiently before TAC reported decreased maladaptive LV hypertrophy and fibrosis (23). Taken together, these data suggest that AMPK is a nexus linking myocardial energetic with LV remodeling and thus a target for early detection and prevention of LV hypertrophy and HF.

Although it has been well-recognized that HP blunts LV hypertrophy and improves cardiac function (6, 7), the process of LV morphological and functional changes in HP mice has been sparsely investigated. We in this study found that short-term variations of loading conditions (TAC 3d, De-TAC 4d) did not alter morphological appearance. However, ERK1 and ERK2, which are central mediators of LV hypertrophy (24), underwent notable changes during the course, providing evidence that hypertrophic response at a post-translational level is not irreversible. Coincidently, we previously demonstrated that preconditioning with Angiotensin II for a short time prevents the subsequent damage of Angiotensin II in cultured endothelial cells, and the protection was abrogated by an ERK inhibitor PD98059 (25), recapitulating the involvement of ERK1/2 in the cardioprotective role of HP. Moreover, ERK1/2 may contribute to the improvement of cardiac energetics induced by HP, since a recent study reports that AMPK stimulation inhibits cardiac hypertrophy through ERK1/2 (26). Since ERK1/2 and AMPK are hub genes involved in coordinations of cell growth, autophagy, and metabolism, more in-depth studies associated with cardiac apoptosis, autophagy, etc., are warranted.

### Limitations

We in this study used β*-actin* as an internal control for normalization of mRNA expression as described previously (7, 27), for it is stably expressed in murine left ventricles subjected to pressure overload (7, 18, 27, 28). However, as the Minimum Information for Publication of Quantitative Real-Time PCR Experiments (MIQE) guidelines for qPCR suggest, two or multiple validated housekeeping genes should be used to ensure the reliability of qPCR data (29). We thus further performed a stability test across our samples and found that the relative level of *GAPDH* is almost equal to that of β*-actin* in our samples (Supplementary Figure 3).

In summary, we for the first time depicted that HP improves energetics, morphology, and function in the pressure overloaded heart. We also demonstrated metabolic changes occurred at early stages before the development of hypertrophy and dysfunction. The time course of events makes it possible that early metabolic abnormalities could serve as antecedent indicators and targets for hypertension-induced LV hypertrophy and HF.

## Data Availability Statement

The original contributions presented in the study are included in the article/Supplementary Materials, further inquiries can be directed to the corresponding author/s.

## Ethics Statement

The animal study was reviewed and approved by Animal Care and Use Committee of Zhongshan Hospital, Fudan University.

## Author Contributions

JW, JL, JH, JYo, and YZ: conception, design, data analysis, and interpretation. JW, JYo, PY, GZ, SW, JYu, XY, JG, and YZ: administrative support. JW, JL, JH, JYo, ZD, LM, FD, RX, XL, and JYu: collection and assembly of data. JW: manuscript writing. All authors wrote first draft, read, amended the draft, and final approval of manuscript.

## Conflict of Interest

The authors declare that the research was conducted in the absence of any commercial or financial relationships that could be construed as a potential conflict of interest.

## References

[1] Badolia R Ramadurai DKA Abel ED Ferrin P Taleb I Shankar TS The role of non-glycolytic glucose metabolism in myocardial recovery upon mechanical unloading and circulatory support in chronic heart failure. Circulation. (2020) 142:259–74. 10.1161/CIRCULATIONAHA.119.044452 32351122 PMC7380956

[2] Wu J Dai F Li C Zou Y. Gender differences in cardiac hypertrophy. J Cardiovasc Transl Res. (2020) 13:73–84. 10.1007/s12265-019-09907-z 31418109

[3] Zou Y Akazawa H Qin Y Sano M Takano H Minamino T. Mechanical stress activates angiotensin II type 1 receptor without the involvement of angiotensin II. Nat Cell Biol. (2004) 6:499–506. 10.1038/ncb1137 15146194

[4] You J Wu J Zhang Q Ye Y Wang S Huang J. Differential cardiac hypertrophy and signaling pathways in pressure versus volume overload. Am J Physiol Heart Circ Physiol. (2018) 314:H552–62. 10.1152/ajpheart.00212.2017 29196344

[5] Byrne NJ Levasseur J Sung MM Masson G Boisvenue J Young ME. Normalization of cardiac substrate utilization and left ventricular hypertrophy precede functional recovery in heart failure regression. Cardiovasc Res. (2016) 110:249–57. 10.1093/cvr/cvw051 26968698 PMC4836630

[6] Wei X Wu B Zhao J Zeng Z Xuan W Cao S. Myocardial hypertrophic preconditioning attenuates cardiomyocyte hypertrophy and slows progression to heart failure through upregulation of S100A8/A9. Circulation. (2015) 131:1506–17. 10.1161/CIRCULATIONAHA.114.013789 25820336 PMC4415966

[7] Huang J Wu J Wang S You J Ye Y Ding Z Asound biomicroscopy validation of a murine model of cardiac hypertrophic preconditioning: comparison with a hemodynamic assessment. Am J Physiol Heart Circ Physiol. (2017) 313:H138–48. 10.1152/ajpheart.00004.2017 28455286

[8] Li J Kemp BA Howell NL Massey J Minczuk K Huang Q. Metabolic changes in spontaneously hypertensive rat hearts precede cardiac dysfunction and left ventricular hypertrophy. J Am Heart Assoc. (2019) 8:e010926. 10.1161/JAHA.118.010926 30764689 PMC6405673

[9] Ritterhoff J Young S Villet O Shao D Neto FC Bettcher LF. Metabolic remodeling promotes cardiac hypertrophy by directing glucose to aspartate biosynthesis. Circ Res. (2020) 126:182–96. 10.1161/CIRCRESAHA.119.315483 31709908 PMC8448129

[10] Hamirani YS Kundu BK Zhong M McBride A Li Y Davogustto GE. Noninvasive detection of early metabolic left ventricular remodeling in systemic hypertension. Cardiology. (2016) 133:157–62. 10.1159/000441276 26594908 PMC4677787

[11] Lai L Leone TC Keller MP Martin OJ Broman AT Nigro J. Energy metabolic reprogramming in the hypertrophied and early stage failing heart: a multisystems approach. Circ Heart Fail. (2014) 7:1022–31. 10.1161/CIRCHEARTFAILURE.114.001469 25236884 PMC4241130

[12] You J Wu J Jiang G Guo J Wang S Li L. Olmesartan attenuates cardiac remodeling through DLL4/notch1 pathway activation in pressure overload mice. J Cardiovasc Pharmacol. (2013) 61:142–51. 10.1097/FJC.0b013e31827a0278 23188126

[13] Constantinides C Murphy K. Molecular and integrative physiological effects of isoflurane anesthesia: the paradigm of cardiovascular studies in rodents using magnetic resonance imaging. Front Cardiovasc Med. (2016) 3:23. 10.3389/fcvm.2016.00023 27525256 PMC4965459

[14] Wu J You J Wang X Wang S Huang J Xie Q. Left ventricular response in the transition from hypertrophy to failure recapitulates distinct roles of Akt, β-arrestin-2, and CaMKII in mice with aortic regurgitation. Ann Transl Med. (2020) 8:219–7. 10.21037/atm.2020.01.51 32309366 PMC7154424

[15] Wu J Bu L Gong H Jiang G Li L Ma H. Effects of heart rate and anesthetic timing on high-resolution echocardiographic assessment under isoflurane anesthesia in mice. J Ultrasound Med. (2010) 29:1771–8. 10.7863/jum.2010.29.12.1771 21098849

[16] Razeghi P Young ME Alcorn JL Moravec CS Frazier OH Taegtmeyer H. Metabolic gene expression in fetal and failing human heart. Circulation. (2001) 104:2923–31. 10.1161/hc4901.100526 11739307

[17] Satoh M Nomura S Harada M Yamaguchi T Ko T Sumida T. High-throughput single-molecule RNA imaging analysis reveals heterogeneous responses of cardiomyocytes to hemodynamic overload. J Mol Cell Cardiol. (2019) 128:77–89. 10.1016/j.yjmcc.2018.12.018 30611794

[18] Sano M Minamino T Toko H Miyauchi H Orimo M Qin Y. p53-induced inhibition of Hif-1 causes cardiac dysfunction during pressure overload. Nature. (2007) 446:444–8. 10.1038/nature05602 17334357

[19] Bairwa SC Parajuli N Dyck JR. The role of AMPK in cardiomyocyte health and survival. Biochim Biophys Acta. (2016) 1862:2199–210. 10.1016/j.bbadis.2016.07.001 27412473

[20] Peterzan M Lygate C Neubauer S Rider O. Metabolic remodeling in hypertrophied and failing myocardium: a review. Am J Physiol Heart Circ Physiol. (2017) 313:H597–616. 10.1152/ajpheart.00731.2016 28646030

[21] Gelinas R Mailleux F Dontaine J Bultot L Demeulder B Ginion A. AMPK activation counteracts cardiac hypertrophy by reducing O-GlcNAcylation. Nat Commun. (2018) 9:374. 10.1038/s41467-017-02795-4 29371602 PMC5785516

[22] Shen C Wang C Han S Wang Z Dong Z Zhao X. Aldehyde dehydrogenase 2 deficiency negates chronic low-to-moderate alcohol consumption-induced cardioprotecion possibly via ROS-dependent apoptosis and RIP1/RIP3/MLKL-mediated necroptosis. Biochim Biophys Acta Mol Basis Dis. (2017) 1863:1912–8. 10.1016/j.bbadis.2016.11.016 27840306

[23] Nam DH Kim E Benham A Park HK Soibam B Taffet GE. Transient activation of AMPK preceding left ventricular pressure overload reduces adverse remodeling and preserves left ventricular function. FASEB J. (2019) 33:711–21. 10.1096/fj.201800602R 30024790 PMC6355077

[24] Zhou N Li L Wu J Gong H Niu Y Sun A. Mechanical stress-evoked but angiotensin II-independent activation of angiotensin II type 1 receptor induces cardiac hypertrophy through calcineurin pathway. Biochem Biophys Res Commun. (2010) 397:263–9. 10.1016/j.bbrc.2010.05.097 20580688

[25] Guan A Zou Y Gong H Niu Y Ye Y Jia J. AngiotensinII preconditioning promotes angiogenesis in vitro via ERKs phosphorylation. J Biomed Biotechnol. (2012) 2012:737134. 10.1155/2012/737134 22500105 PMC3303689

[26] Meng R Pei Z Zhang A Zhou Y Cai X Chen B. AMPK activation enhances PPARalpha activity to inhibit cardiac hypertrophy via ERK1/2 MAPK signaling pathway. Arch Biochem Biophys. (2011) 511:1–7. 10.1016/j.abb.2011.04.010 21530483

[27] Schuman ML Landa MS Toblli JE Peres Diaz LS Alvarez AL Finkielman S. Cardiac thyrotropin-releasing hormone mediates left ventricular hypertrophy in spontaneously hypertensive rats. Hypertension. (2011) 57:103–9. 10.1161/HYPERTENSIONAHA.110.161265 21135357

[28] Fang X Robinson J Wang-Hu J Jiang L Freeman DA Rivkees SA. cAMP induces hypertrophy and alters DNA methylation in HL-1 cardiomyocytes. Am J Physiol Cell Physiol. (2015) 309:C425–36. 10.1152/ajpcell.00058.2015 26224577 PMC4572371

[29] Bustin SA Benes V Garson JA Hellemans J Huggett J Kubista M. The MIQE guidelines: minimum information for publication of quantitative real-time PCR experiments. Clin Chem. (2009) 55:611–22. 10.1373/clinchem.2008.112797 19246619

