# Supplementary material for: Variations in Energy Metabolism Precede Alterations in Cardiac Structure and Function in Hypertrophic Preconditioning
Source: Front Cardiovasc Med. 2020 Dec 11;7:602100. doi: 10.3389/fcvm.2020.602100 (PMC7793816; doi:10.3389/fcvm.2020.602100)
Supplement: Supplementary file 1 [file Table_1.DOCX]

**Supplemental Table.** Primers used in real-time quantitative PCR.

| Gene | Sequence (5'-3') |
| --- | --- |
| *anp* | Forward: CTGCTTCGGGGGTAGGATTG |
|  | Reverse: GCTCAAGCAGAATCGACTGC |
| *bnp* | Forward: GAGGTCACTCCTATCCTCTGG |
|  | Reverse: GCCATTTCCTCCGACTTTTCTC |
| *col1α1* | Forward: AGCACGTCTGGTTTGGAGAG |
|  | Reverse: GACATTAGGCGCAGGAAGGT |
| *col3α1* | Forward: ACGTAAGCACTGGTGGACAG |
|  | Reverse: CAGGAGGGCCATAGCTGAAC |
| *glut4* | Forward:  GCCCCATTCCCTGGTTCATT |
|  | Reverse: GACCCATAGCATCCGCAACA |
| *pdk4* | Forward:  GAATGCCCCTTTGGCTGGT |
|  | Reverse: TAGCGTCTGTCCCATAACCTG |
| *mcad* | Forward: GAAGCCACGAAGTATGCCCT |
|  | Reverse:  CCTTCATCGCCATTTCTGCG |
| *pgc-1α* | Forward: TGTGTGCTGTGTGTCAGAGT |
|  | Reverse: ACCAGAGCAGCACACTCTATG |
| *mcd* | Forward: CATGTGGCTCTGACTGGTGA |
|  | Reverse: CTCTCCTCGGTTTCAGTCGG |
| *ppara* | Forward: GGAAAGACCAGCAACAACCC |
|  | Reverse: GAATCGGACCTCTGCCTCTT |
| *actb* | Forward:  CACTGTCGAGTCGCGTCC |
|  | Reverse: TCATCCATGGCGAACTGGTG |
