# Supplementary material for: Variations in Energy Metabolism Precede Alterations in Cardiac Structure and Function in Hypertrophic Preconditioning
Source: Front Cardiovasc Med. 2020 Dec 11;7:602100. doi: 10.3389/fcvm.2020.602100 (PMC7793816; doi:10.3389/fcvm.2020.602100)
Supplement: Supplementary file 2 [file Image_1.PDF]

Supplemental Figure S1

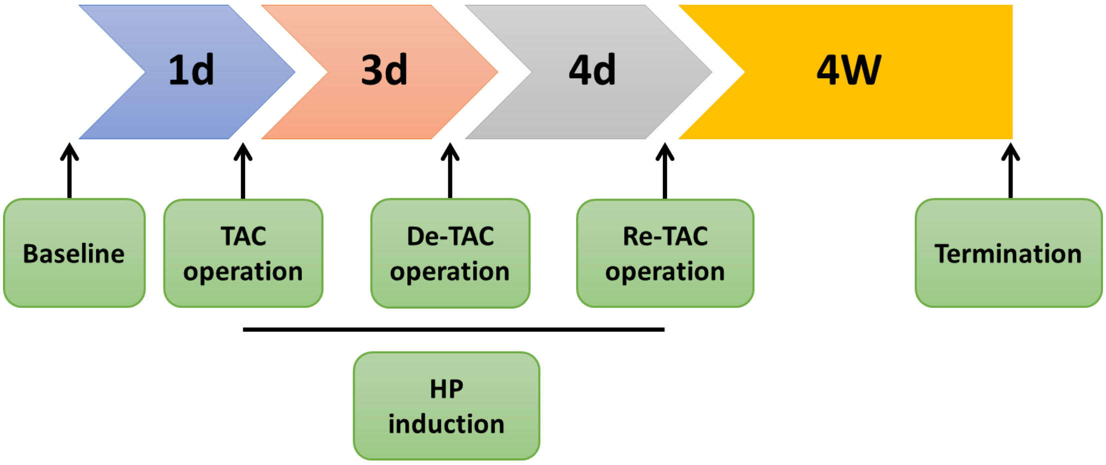

Schematic of the process of Re-TAC 4W. Serial assessments were performed at Baseline (1 day before operation), 3 days after transverse aortic constriction (TAC 3d), 4 days after aortic de-banding (De-TAC 4d), and 4 weeks after re-banding (Re-TAC 4W). The HP induced cardioprotection is generated by de-banding of short-term TAC, which protects the heart against hypertrophic responses induced by subsequent re-constriction (Re-TAC) in mice.

Supplemental Figure S2

A

B

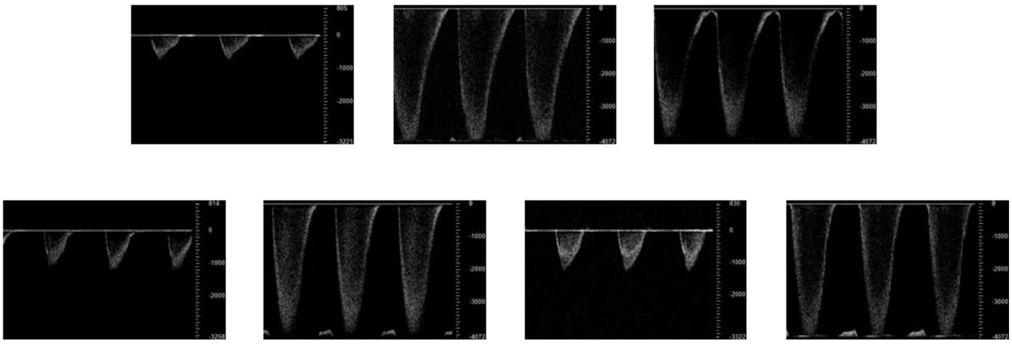

Series of pulse-wave Doppler images showing peak flow velocity at aortic banding sites. (A): from left to right, Sham, TAC 4W, and Re-TAC 4W mice. Striking increases of peak flow velocity at the aortic banding site (PVb) suggest successful aortic banding in TAC 4W and Re-TAC 4W mice. (B) from left to right, evaluation time points of Baseline, TAC 3d, De-TAC 4d, Re-TAC 4W. PVb is successfully elevated at TAC 3d and Re-TAC 4W, and reduced to Baseline level at De-TAC 4d.

Supplemental Figure S3

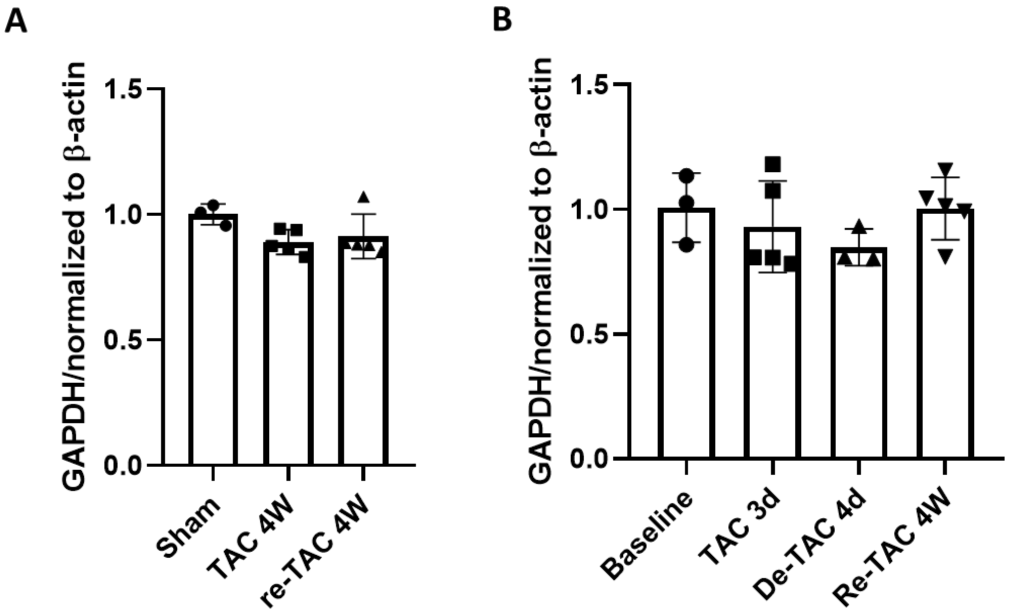

Investigation of the correlations of *GAPDH* and *β-actin*. The relative mRNA expression level of *GAPDH* is almost equal to that of *β-actin*. (A): from left to right, heart tissues from Sham, TAC 4W, and Re-TAC 4W mice. (B) from left to right, heart tissues from Baseline, TAC 3d, De-TAC 4d, Re-TAC 4W mice.
